# Knowledge and Awareness of Screening for Children With Cryptorchidism in the Al-Qunfudhah Governorate, Saudi Arabia

**DOI:** 10.7759/cureus.59770

**Published:** 2024-05-06

**Authors:** Medhat Taha, Saja Ahmed Alqarni, Fatimah Mohammed Alshamrani, Enas Mousa Alqarni, Amna Ahmad Almathami, Maha Ahmad Almathami, Norah Ali Alamri

**Affiliations:** 1 Department of Anatomy, Umm Al-Qura University, Al-Qunfudhah, SAU; 2 Department of Medicine, Umm Al-Qura University, Al-Qunfudhah, SAU

**Keywords:** attitudes, knowledge, al-qunfudah, children, undescended testis

## Abstract

Background

Undescended testis is a common pediatric surgical presentation condition with potential long-term consequences if left untreated. It is characterized by the failure of one or both testes to descend into the scrotum. This study aims to measure and enhance awareness and knowledge about undescended testis through comprehensive medical research and provide evidence-based recommendations.

Objective

The objective of this study was to evaluate participants' knowledge regarding undescended testes and assess the level of interest and awareness among individuals and parents about the importance of early examination and treatment.

Methods

It is a cross-sectional, nationwide study targeting the population of Al-Qunfudhah. The study was conducted in December 2023 using a validated questionnaire distributed through social media platforms.

Results

The study analyzed data from 459 participants to assess their knowledge and attitudes regarding undescended testis. Participants' knowledge was evaluated. In general, the mean ± SD score of knowledge was 3.61 ± 2.33. Higher education level, occupation, and having children were associated with greater knowledge. Attitudes were measured. The mean attitudes score was 2.37 ± 1.58. Higher education level, occupation, and marital status influenced attitudes.

Conclusion

This study provides valuable insights into the knowledge and attitudes of individuals regarding undescended testis. Participants displayed moderate levels of knowledge and positive attitudes, with educational attainment and occupation playing significant roles. These findings highlight the importance of targeted educational interventions to improve awareness and promote positive attitudes toward undescended testis.

## Introduction

The awareness of the undescended testis (UDT) screening is crucial for early detection, treatment, and prevention of complications. The primary causes of UDT treatment are elevated risks of infertility, testicular cancer, torsion, and/or associated inguinal hernia, and can significantly affect a person's quality of life [1-5]. Previous research indicates that there are large observed numbers of delayed presentations of children diagnosed with UDT in our community [6].

It is crucial to have a comprehensive understanding of UDT screening by parents who want to have children to develop effective interventions to prevent late presentation and to minimize the risk of infertility and malignancy which are complications associated with UDT.

The most prevalent congenital urogenital abnormality in boys is undescended testicles (UDT), medically termed cryptorchidism [7,8]. It is referred to as the failure of the testis to descend into the usual scrotal position on one or both sides by the time of birth [9]. Until now the cause of UDT is unknown [10]. It is estimated that the incidence of UDT in full-term infants or those weighing more than 2.5 kg is 4.6%, whereas the incidence in preterm infants or those weighing less than 2.5 kg varies from 1.1% to 45.3%; in a previous Saudi study, 22.4% of cases were diagnosed immediately after birth, 21.4% within the first three months of life, 12.4% between three and six months, 12.1% between seven months and a year, and 31.7% over a year [6,7]. The normal spontaneous testicular descent occurs in the first six months of life due to an elevated level of gonadotropins and androgenic hormones; after that, the possibility of a spontaneous descent will decrease [11,12,3]. Testicular descent is necessary for spermatogenesis [10]. The importance of early treatment and diagnosis lies in the prevention of later complications. According to numerous international guidelines, surgical correction (orchidopexy) is recommended between the ages of 6 and 18 months and ideally completed before one year of age. Meeting these timing recommendations is crucial for preserving male fertility in adulthood, primarily due to potential issues with semen quality [13,14].

In Saudi Arabia, the median age at the time of surgical correction of UDT was found to be twice that of the international recommendations, exceeding the recommended surgery time [13]. A study done in Saudi Arabia in 2023 found that a total of 2360 people were enrolled. Over half didn't know about UDT. The age of the presentation was not known by 48.5% of the participants [7]. This is primarily due to the late referral age, the lengthy waiting period for elective surgery [9], and the lack of awareness of the existence of this defect and delayed diagnosis.

There is a need for further study on the awareness of UDT screening in the general population in the Al-Qunfudhah governorate due to limited research in this area. The objective of this study was to examine the level of awareness for undescended testis screening in the Al-Qunfudhah governorate by gathering the number of the general population who are aware of the importance of early screening of UDT for their children. Additionally, the research aimed to investigate the knowledge about UDT and its treatment and complications in the Al-Qunfudhah governorate.

## Materials and methods

Study design, allocation, and timing 

This was a cross-sectional observational study done in the Al-Qunfudhah region of Saudi Arabia in December 2023 after getting approval (approval no. HAPO-02-K-012-2023-12-1935) from the Biomedical Research Ethics Committee of Umm Al-Qura University, Al-Qunfudhah, Saudi Arabia.

Study population (participants)

The inclusion criteria constituted the general population living in the Al-Qunfudhah region of Saudi Arabia who agreed to participate in the study. They had to be adults, meaning they had to be greater than or equal to 18 years of age. They had to have access to social media platforms. Exclusion criteria include any participant who did not live in the Al-Qunfudhah region of Saudi Arabia.

Study tool and data collection

A cross-sectional observational electronic questionnaire (see Appendices) was used to assess the knowledge and awareness of people about screening in children with UDT in the Al-Qunfudhah governorate, Kingdom of Saudi Arabia. The survey was created in Google Forms (Google LLC, Mountain View, California, United States) and distributed through social media platforms (e.g., Twitter (X Corp., San Francisco, California, United States), Instagram (Meta Platforms, Inc., Menlo Park, California, United States), and WhatsApp (Meta Platforms, Inc., Menlo Park, California, United States)). The result was 533 participants. The questionnaire consisted of 21 questions and was divided into three sections. The first part was about the respondents' demographic information (e.g., gender, age, material status, educational level, occupation, and whether they had children). In the second part, the participants were asked 10 questions about UDT, and the purpose of these questions was to find out if they had sufficient experience and information and also to evaluate the extent of their awareness and knowledge about UDT. (At which age does UDT arise? Does a UDT affect testicular function? Does UDT have any complications? What is the treatment for UDT? What is the optimum time for surgery? Is there any benefit from early treatment? Do you think testicular atrophy is a potential harm of delaying the intervention? Do you think infertility is a potential harm of delaying the intervention? Do you think testicular torsion is a potential harm of delaying the intervention? Do you think malignancy risk is a potential harm of delaying the intervention?) 

Finally, in the third part of the questionnaire, questions were asked to know the extent of interest and awareness of individuals and parents about the issue of undescended testicles. The aim of these questions was to evaluate community awareness and provide recommendations and advice on the importance of early treatment for children with undescended testicles. Among these questions are: Do you advise other parents to have their children examined to ensure that the testicle is not descended? What factors might influence your recommendation to other parents to have their child screened for undescended testicles? How important is early detection and treatment of undescended testicles to the child’s overall health and well-being? If you have a friend or family member whose child suffers from undescended testicles, would you encourage him to seek medical evaluation and treatment? How confident are you in your knowledge about undescended testicles and their possible consequences?

Calculation of the knowledge and attitudes scores

Participants’ knowledge in the current study was assessed based on eight items. An overall knowledge score was calculated for each participant by assigning a score of 1 to correct answers and a score of 0 to incorrect answers or responses such as "do not know" for each of the eight knowledge items related to UDT. These scores were then summed up to derive the participant's total knowledge score, which ranged from 0 to 8. A higher score indicated a greater level of knowledge regarding UDT, with a maximum achievable score of 8 representing perfect knowledge. Regarding participants’ attitudes, we used four items to evaluate participants’ perceptions regarding UDT. One of the four items was a multiple-choice item, with four available choices indicating positive attitudes. Therefore, we had a total of seven choices reflecting positive attitudes. A confirmed response to a positive attitude item was assigned 1 and other responses were assigned zero. Therefore, an overall positive attitudes score ranged between 0 and 7, with higher scores indicating more positive attitudes.

Statistical analysis 

The statistical analysis was conducted using RStudio software (R version 4.3.1, R Foundation, Vienna, Austria). Descriptive statistics were used to summarize the sociodemographic characteristics of the participants, attitudes, and knowledge regarding UDT. Frequencies and percentages were calculated for categorical variables, while means and standard deviations were calculated for continuous variables. Inferential analysis was performed to identify associations, knowledge, and attitudes regarding UDT. Inferential tests included the Wilcoxon rank-sum test or the Kruskal-Wallis test. Multivariable regression analysis was conducted to identify predictors of knowledge and attitudes by including the significantly associated variables from the inferential analysis as independent variables. We used the knowledge and attitudes scores as dependent variables (each score in a separate model). Beta coefficients along with 95% confidence intervals were reported for the regression analysis. Variables with a p-value < 0.05 were considered statistically significant.

## Results

Sociodemographic characteristics

Initially, we collected data from 533 respondents. However, we excluded 10 records of those who refused to participate and 64 records of respondents aged < 18 years. Therefore, we analyzed the responses of 459 participants in the current study. Among them, the majority were female (n=243, 52.9%). Regarding age distribution, the largest proportion fell within the 18 to 25 years bracket (n=142, 30.9%). In terms of marital status, married individuals constituted the highest proportion (n=150, 32.7%). Bachelor's degree holders comprised the largest educational group (n=161, 35.1%), and among occupations, the employed category had the highest representation (n=158, 34.4%). Moreover, a considerable majority resided in urban areas (n=272, 59.3%). Less than half of the sample had children (n=207, 45.8%) (Table 1).

**Table 1:** Sociodemographic characteristics *The variable had seven missing records

Characteristic	N (%)
Gender	
Male	216 (47.1%)
Female	243 (52.9%)
Age (years)	
18 to 25	142 (30.9%)
26 to 35	132 (28.8%)
36 to 45	95 (20.7%)
46 to 55	50 (10.9%)
56 or more	40 (8.7%)
Marital status	
Single	124 (27.0%)
Married	150 (32.7%)
Divorced	127 (27.7%)
Widowed	58 (12.6%)
Educational level	
Less than high school	28 (6.1%)
High school	94 (20.5%)
Diploma	106 (23.1%)
Bachelor	161 (35.1%)
Post-graduate	70 (15.3%)
Occupation	
Student	98 (21.4%)
Employed	158 (34.4%)
Non-employed	145 (31.6%)
Retired	58 (12.6%)
City of residence	
Urban	272 (59.3%)
Rural	187 (40.7%)
Do you have children?*	207 (45.8%)

Knowledge regarding UDT and the associated factors

In assessing awareness and knowledge regarding UDT, it was found that 42.5% of participants correctly identified that it arises since birth (n=195). Regarding its impact on testicular function, 173 participants (37.7%) recognized that UDT does affect testicular function. Similarly, 172 participants (37.5%)acknowledged the existence of complications associated with UDT (n=172, 37.5%). Notably, 40.1% correctly identified surgical intervention as the treatment for UDT (n=184). Concerning the optimum time for surgery, 34.9% correctly indicated the period between six months to one year (n=160). Moreover, a substantial proportion (n=173, 37.7%) recognized the benefit of early treatment. Additionally, a significant majority (n=290, 63.2%) acknowledged testicular torsion as a potential harm of delaying intervention, while 67.3% (n=309) recognized malignancy risk as another potential harm of delaying intervention (Table 2).

**Table 2:** Awareness and knowledge regarding undescended testis *An asterisk indicates a correct response

Characteristic	N (%)
At which age does undescended testis arise?	
Since birth*	195 (42.5%)
Adolescent age	169 (36.8%)
Do not know	95 (20.7%)
Does undescended testis affect the testicular function?	
No	159 (34.6%)
Yes*	173 (37.7%)
Do not know	127 (27.7%)
Does undescended testis have any complications?	
No	134 (29.2%)
Yes*	172 (37.5%)
Do not know	153 (33.3%)
What is the treatment of undescended testis?	
Oral medication	67 (14.6%)
Hormone therapy	87 (19.0%)
Surgical intervention*	184 (40.1%)
Does not need treatment	45 (9.8%)
Do not know	76 (16.6%)
What is the optimum time for surgery?	
After birth	64 (13.9%)
First five months	97 (21.1%)
Six months to one year*	160 (34.9%)
One year and more	67 (14.6%)
Do not know	71 (15.5%)
Is there any benefit from early treatment?	
No	163 (35.5%)
Yes*	173 (37.7%)
Do not know	123 (26.8%)
Do you think testicular torsion is a potential harm of delaying the intervention?	
No	169 (36.8%)
Yes*	290 (63.2%)
Do you think malignancy risk is a potential harm of delaying the intervention?	
No	150 (32.7%)
Yes*	309 (67.3%)

**Figure 1:**
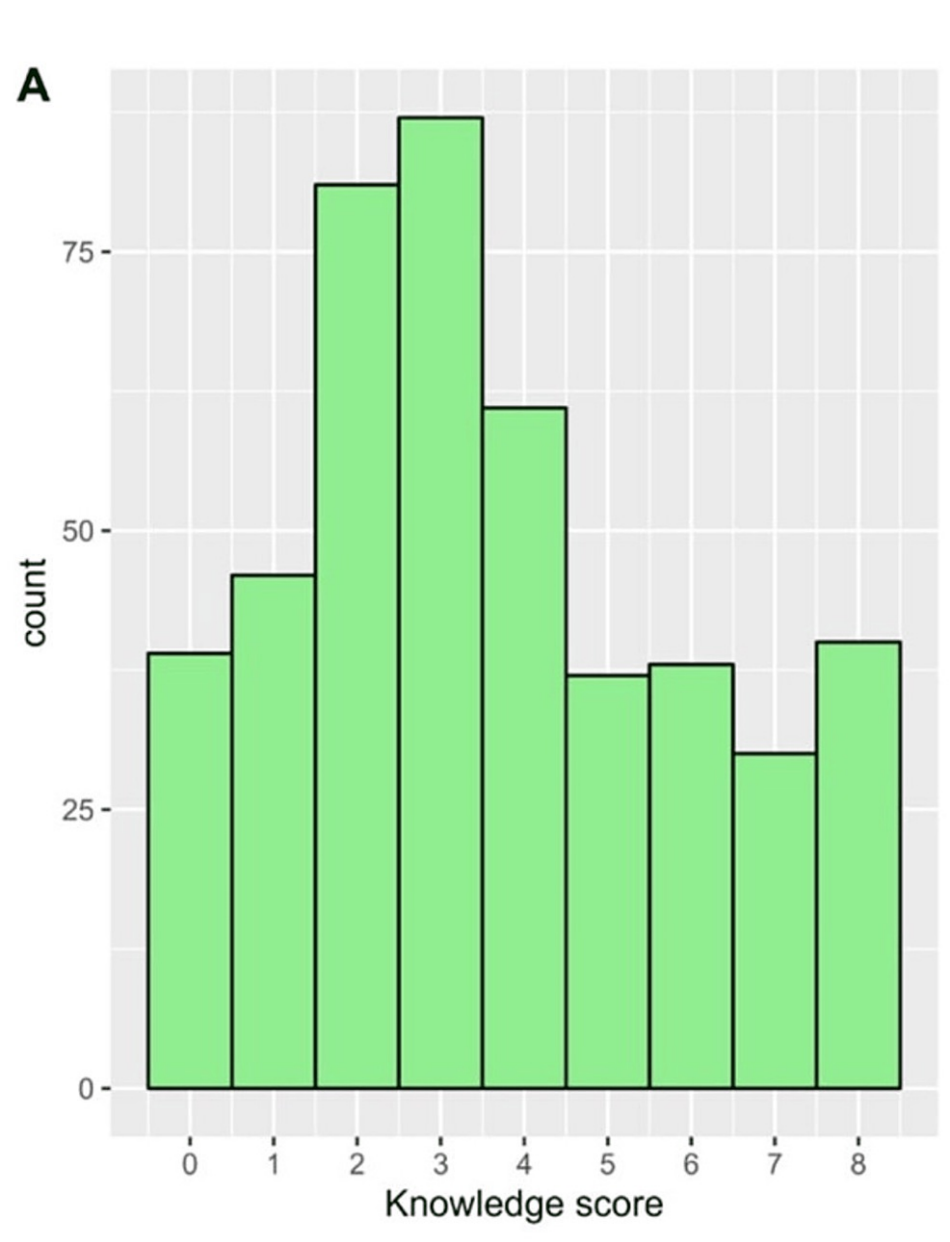
The distribution of the scores of knowledge regarding undescended testis

In general, the mean ± SD score of knowledge was 3.61 ± 2.33. The distribution of the knowledge score is depicted in Figure 1. On the multivariable regression analysis, participants with a higher educational level, specifically those with a bachelor's degree (beta = 1.17, 95% CI: 0.27 to 2.06, p = 0.011) or post-graduate education (beta = 1.17, 95% CI: 0.12 to 2.22, p = 0.030), demonstrated significantly greater knowledge compared to those with less education.

Additionally, occupation played a significant role, with non-employed individuals (beta = -1.11, 95% CI: - 1.77 to -0.45, p = 0.001) and retired individuals (beta = -1.66, 95% CI: -2.59 to -0.73, p < 0.001) showing lower levels of knowledge compared to students. Furthermore, participants who had children exhibited higher knowledge levels (beta = 0.73, 95% CI: 0.27 to 1.18, p = 0.002) compared to those without children (Table 3).

**Table 3:** Factors and predictors of knowledge regarding undescended testis CI = Confidence Interval; SD: standard deviation; NA: non-applicable Data are represented as mean ± SD for the inferential analysis and beta coefficients (95% confidence intervals) for the multivariable regression. Bold p-values indicated statistical significance at p < 0.05

Inferential analysis			Multivariable regression		
Characteristic	Mean ± SD	p-value	Beta	95% CI	p-value
Gender		0.769			
Male	3.54 ± 2.13		NA	NA	NA
Female	3.67 ± 2.49		NA	NA	NA
Age (years)		0.068			
18 to 25	3.99 ± 2.45		NA	NA	NA
26 to 35	3.39 ± 2.11		NA	NA	NA
36 to 45	3.59 ± 2.35		NA	NA	NA
46 to 55	3.74 ± 2.18		NA	NA	NA
56 or more	2.85 ± 2.50		NA	NA	NA
Marital status		0.009			
Single	4.22 ± 2.65		Reference	Reference	
Married	3.64 ± 2.17		-0.34	-0.97, 0.29	0.292
Divorced	3.27 ± 1.99		-0.57	-1.23, 0.09	0.091
Widowed	2.97 ± 2.38		-0.73	-1.62, 0.15	0.103
Educational level		<0.001			
Less than high school	3.61 ± 2.10		Reference	Reference	
High school	3.16 ± 1.82		-0.10	-1.05, 0.86	0.845
Diploma	2.91 ± 1.66		-0.08	-1.05, 0.89	0.871
Bachelor	4.37 ± 2.57		1.17	0.27, 2.06	0.011
Post-graduate	3.53 ± 2.79		1.17	0.12, 2.22	0.030
Occupation		<0.001			
Student	4.43 ± 2.56		Reference	Reference	
Employed	3.95 ± 2.25		-0.39	-1.03, 0.25	0.231
Non-employed	3.06 ± 1.91		-1.11	-1.77, -0.45	0.001
Retired	2.67 ± 2.45		-1.66	-2.59, -0.73	<0.001
City of residence		0.005			
Urban	3.86 ± 2.28		Reference	Reference	
Rural	3.24 ± 2.34		-0.36	-0.79, 0.06	0.093
Do you have children?		0.030			
No	3.44 ± 2.47		Reference	Reference	
Yes	3.81 ± 2.15		0.73	0.27, 1.18	0.002
Unknown	7				

**Figure 2:**
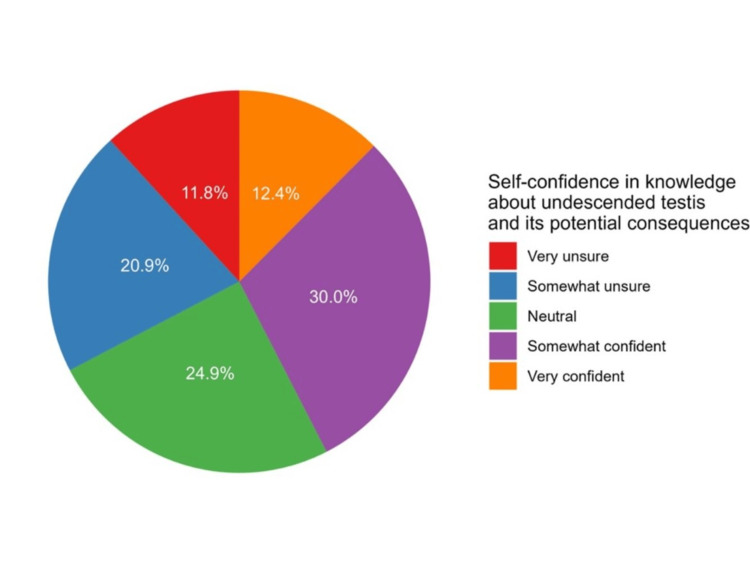
Participants’ responses regarding their self-confidence in knowledge about undescended testis and its potential consequences

Notably, approximately 24.9% of participants (n=114) expressed uncertainty, 11.8% (n=53) indicated they were "very unsure," and 20.9% (n=94) felt "somewhat unsure". Conversely, confidence levels were notable, with 30.0% of participants feeling "somewhat confident" (n=135) and 12.4% reporting feeling "very confident" (n=56) about their knowledge regarding UDT and its potential consequences (Figure 2).

Attitudes regarding UDT

Regarding attitudes regarding undescended testis, 44.0% of participants expressed a positive attitude by indicating that they would definitely recommend other parents to have their child screened for undescended testis (n=202). Factors influencing this recommendation included the availability of effective treatment options (n=224, 48.8%), awareness of potential long-term consequences of untreated undescended testis (n=163, 35.5%), personal experience or knowledge of someone with UDT (n=166, 36.2%), and advice from healthcare professionals (n=119, 25.9%). Furthermore, 24.8% of participants considered early detection and treatment of UDT as extremely important for a child's overall health and well-being (n=114). Additionally, 22.0% stated that they would encourage a friend or family member to seek medical evaluation and treatment for their child with UDT without hesitation (n=101) (Table 4).

**Table 4:** Attitudes regarding undescended testis *An asterisk indicates a response reflecting positive attitudes

Characteristic	N (%)
Would you recommend other parents to have their child screened for undescended testis?	
No, definitely not	12 (2.6%)
Probably not	32 (7.0%)
Not sure	77 (16.8%)
Probably yes	136 (29.6%)
Yes, definitely*	202 (44.0%)
Factors that influence the recommendation to other parents for screening their child for undescended testis	
Potential long-term consequences of untreated undescended testis*	163 (35.5%)
Availability of effective treatment options*	224 (48.8%)
Personal experience or knowledge of someone with undescended testis*	166 (36.2%)
Advice from healthcare professionals*	119 (25.9%)
Others	33 (7.2%)
How important do you think early detection and treatment of undescended testis is for a child's overall health and well-being?	
Not important at all	25 (5.4%)
Slightly important	137 (29.8%)
Moderately important	183 (39.9%)
Very important	0 (0.0%)
Extremely important*	114 (24.8%)
If you had a friend or family member whose child had undescended testis, would you encourage them to seek medical evaluation and treatment?	
No, definitely not	17 (3.7%)
No, probably not	52 (11.3%)
Not sure	137 (29.8%)
Yes, but with some reservations	152 (33.1%)
Yes, without hesitation*	101 (22.0%)

**Figure 3:**
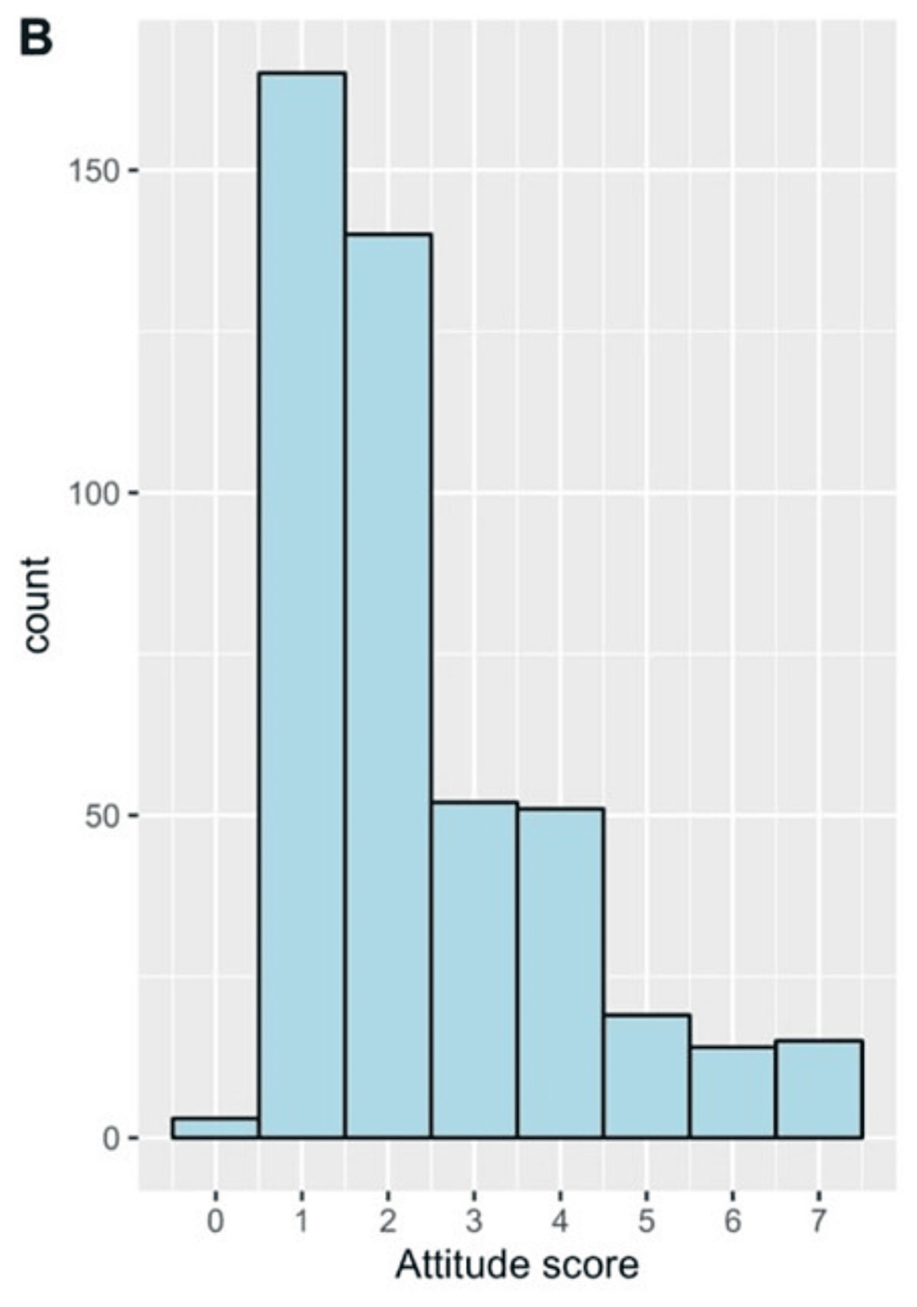
The distribution of the scores of attitudes (B) regarding undescended testis

The mean attitudes score was 2.37 ± 1.58. The frequency distribution of the positive attitudes score is shown in Figure 3.

In the multivariable regression analysis concerning factors and predictors of attitudes regarding UDT, results showed that participants with higher educational attainment, specifically those with a diploma (beta = 0.68, 95% CI: 0.03 to 1.32, p = 0.039), bachelor's degree (beta = 1.22, 95% CI: 0.62 to 1.81, p < 0.001), or post-graduate education (beta = 1.56, 95% CI: 0.86 to 2.25, p < 0.001), demonstrated more positive attitudes. Occupation also played a significant role, with non-employed individuals (beta = -1.14, 95% CI: -1.57 to -0.70, p < 0.001), retired individuals (beta = - 1.13, 95% CI: -1.74 to -0.51, p < 0.001), and employed individuals (beta = -0.63, 95% CI: -1.05 to -0.20, p = 0.004) exhibiting less positive attitudes compared to students. Additionally, marital status emerged as a significant predictor, with divorced individuals (beta = -0.56, 95% CI: -0.98 to -0.14, p = 0.010) demonstrating less positive attitudes compared to single individuals (Table 5).

**Table 5:** Factors and predictors of attitudes regarding undescended testis CI = Confidence Interval; SD: standard deviation; NA: non-applicable Data are represented as mean ± SD for the inferential analysis and beta coefficients (95% confidence intervals) for the multivariable regression *The variable had seven missing records

Inferential analysis			Multivariable regression		
Characteristic	Mean ± SD	p-value	Beta	95% CI	p-value
Gender		0.066			
Male	2.20 ± 1.46		NA	NA	NA
Female	2.52 ± 1.66		NA	NA	NA
Age (years)		0.076			
18 to 25	2.68 ± 1.67		NA	NA	NA
26 to 35	2.22 ± 1.47		NA	NA	NA
36 to 45	2.19 ± 1.42		NA	NA	NA
46 to 55	2.34 ± 1.75		NA	NA	NA
56 or more	2.28 ± 1.65		NA	NA	NA
Marital status		<0.001			
Single	3.02 ± 1.79		Reference	Reference	
Married	2.24 ± 1.54		-0.38	-0.78, 0.02	0.065
Divorced	1.96 ± 1.21		-0.56	-0.98, -0.14	0.010
Widowed	2.24 ± 1.51		-0.49	-1.07, 0.08	0.091
Educational level		<0.001			
Less than high school	1.96 ± 0.84		Reference	Reference	
High school	2.01 ± 1.19		0.46	-0.17, 1.09	0.151
Diploma	1.89 ± 1.13		0.68	0.03, 1.32	0.039
Bachelor's	2.83 ± 1.85		1.22	0.62, 1.81	<0.001
Post-graduate	2.70 ± 1.79		1.56	0.86, 2.25	<0.001
Occupation		<0.001			
Student	3.23 ± 1.82		Reference	Reference	
Employed	2.41 ± 1.57		-0.63	-1.05, -0.20	0.004
Non-employed	1.82 ± 1.16		-1.14	-1.57, -0.70	<0.001
Retired	2.21 ± 1.46		-1.13	-1.74, -0.51	<0.001
City of residence		0.605			
Urban	2.42 ± 1.63		NA	NA	NA
Rural	2.30 ± 1.49		NA	NA	NA
Do you have children?*		0.114			
No	2.50 ± 1.63		NA	NA	NA
Yes	2.24 ± 1.52		NA	NA	NA

## Discussion

The objectives of this research were to assess the knowledge and awareness of UDT screening among individuals and parents in the Al-Qunfudhah governorate, Saudi Arabia. Specifically, the study aimed to evaluate participants' understanding of UDT, determine their level of interest in early examination and treatment, and identify factors influencing knowledge and attitudes toward UDT. Additionally, the research sought to provide insights into the importance of targeted educational interventions to enhance awareness and promote positive attitudes towards UDT screening in the local community.

We assessed participants' knowledge because it is critical for recognizing and presenting the illness early. In our study, married individuals comprised the highest percentage of those who were knowledgeable and aware, with 150 participants representing 32.7% of the total sample. Among the professions, the category of workers exhibited the highest representation, with 158 participants accounting for 34.4% of the sample. From these findings, it can be inferred that marital status plays a significant role in awareness and knowledge. Compared to the findings of the Makkah, Saudi Arabia, study [7] and the Hail, Saudi Arabia, study [6], individuals with previous experience exhibited the highest level of knowledge, indicating a clear association between knowledge and experience. Our survey revealed that one-quarter of the participants lacked awareness and expertise, a finding consistent with the Makkah study [7], which identified a lack of early filing software as a prevalent reason for delayed UDT presentation. Similarly, the Hail study [6] found that a delay in transfer to the surgical department was the primary cause.

When comparing the knowledge and attitudes regarding UDT between our study and another study entitled 'Knowledge Level of Undescended Testis in Saudi Arabia: Why Are We Facing Delayed Presentation?' [7], there are several similarities and differences. In both studies, a significant proportion of participants demonstrated limited awareness of UDT. Our study in Saudi Arabia found that over half of the participants had not heard about UDT, while the second study [7] showed that 54.92% of respondents had never heard about it. Similarly, both studies highlighted gaps in knowledge regarding the age of UDT presentation and its treatment modality.

However, there are notable differences in the findings as well. Our study reported that 13.9% of participants had known someone diagnosed with UDT, whereas the second study [7] found that 15.4% of participants had prior experience with UDT. Additionally, while our study identified delayed presentation factors such as a lack of community awareness and parental neglect, the second study did not explicitly address these factors. Regarding surgical intervention, both studies (our study and [7]) found comparable rates of surgery among diagnosed UDT cases. Our study reported that 56% of participants were operated surgically. In the second study, 51.4% of participants underwent surgical intervention after being diagnosed with UDT, while 64.6% of participants had relatives and friends who underwent surgical intervention for the same condition.

To compare with another study entitled “Assessment of Level of Knowledge About Cryptorchidism and Its Complications Among Paternity Age Population in the Western Region of Saudi-Arabia. Cross-Sectional Questionnaire Based Study”[10], in our study, conducted in Saudi Arabia, the mean knowledge score regarding UDT was 3.61 out of 8, with a significant association between educational level and knowledge. In contrast, the other study found a mean knowledge score of 7.5 out of 20, indicating higher overall knowledge among participants. However, both studies observed a significant association between educational level and knowledge about UDT. Regarding the prevalence of UDT, our study reported a prevalence of 13.9% among participants who had known someone diagnosed with UDT, while the second study found a prevalence of 8.4% among its participants. Both studies highlighted gaps in awareness, with a substantial proportion of participants having never heard of UDT. Furthermore, both studies identified misconceptions about UDT among participants. In our study, 42.5% of participants correctly identified that UDT arises since birth, whereas in the second study, 61.8% believed that UDT could be present since birth. Additionally, both studies found that a significant proportion of participants were unaware of the suitable time for surgical intervention for UDT. Overall, while there are differences in the reported prevalence and knowledge levels between the two studies, they both emphasize the need for improved education and awareness regarding UDT among the population.

When comparing the knowledge and attitudes regarding UDT between our study and another study entitled “Evaluation of Children with Undescended Testes Referred to Children’s Medical Center in 5 years [15], we can notice many important differences. The two studies offered complementary perspectives on UDT, shedding light on both public awareness and clinical management practices. Our study delved into the realm of public knowledge and awareness regarding UDT, assessing the understanding of causes, consequences, and treatment options among a sample population. With a substantial sample size of 459 participants, the study revealed moderate awareness levels, influenced significantly by factors such as educational level, occupation, and parenthood status. Meanwhile, the other study focused on the clinical aspects of UDT, evaluating the diagnosis, management, and outcomes of pediatric cases referred to a medical center over a five-year period. By analyzing patient records, the study elucidated trends in age at diagnosis and surgery, types of UDT, incidence of prematurity, and coexisting pathologies. Notably, it identified a high rate of diagnosis by eleven years of age but underscored delay in surgical intervention, with most surgeries performed beyond the optimal age window. Both studies underscored the importance of addressing gaps in public awareness and ensuring timely clinical intervention for UDT. While our Study highlighted the significance of education and occupation in shaping awareness levels, the other study [15] emphasized the need for improved diagnostic practices and collaborative management strategies among healthcare professionals.

Together, these findings contribute to a comprehensive understanding of UDT, advocating for enhanced public education efforts and streamlined clinical protocols to optimize patient outcomes.

This is an important study [16], which aimed to assess whether the management of UDT could be improved with educational updates and a new transferring model among referring providers (RPs) in a hospital setting in China. The study analyzed the age of orchidopexies performed before and after the implementation of educational updates and new transferring models. It found that despite educational efforts, the median age of orchidopexy did not match the target age of 6-12 months. However, there was a statistically significant downward trend in the age of orchidopexy after the intervention, suggesting some improvement in the management of UDT.

This study has several limitations. Firstly, reaching a representative sample of the population in the Al-Qunfudhah governorate through social media platforms may have introduced sampling bias, as individuals without internet access or social media accounts were excluded. Additionally, the reliance on self-reported data may have introduced response bias, as participants may have provided socially desirable answers. Furthermore, cultural and societal norms regarding discussing sensitive topics such as reproductive health could have affected participants' willingness to engage with the questionnaire. Lastly, interpreting the results may have been challenging due to variations in educational backgrounds and levels of health literacy among participants, impacting the accuracy of knowledge and attitudes assessments.

## Conclusions

The management of UDT remains a critical issue in pediatric healthcare, with significant implications for long-term fertility and health outcomes. The goal of recent studies, including ours, has been to improve the timely diagnosis and treatment of UDT to mitigate associated risks. While interventions such as educational updates and new transferring models among healthcare providers have shown promising results in reducing the age at orchidopexy, challenges persist in achieving optimal outcomes.

Our study, along with others, has highlighted the need for continued efforts to streamline referral pathways, increase awareness among healthcare providers, and enhance collaboration between primary care and specialty centers. Despite improvements in certain aspects of UDT management, further research is warranted to address remaining barriers and optimize treatment protocols.

To future researchers, doctors, and parents, we advise prioritizing early detection and timely intervention for UDT cases. It is essential to advocate for comprehensive educational initiatives, standardized referral pathways, and multidisciplinary care approaches to ensure that children with UDT receive timely and appropriate treatment, ultimately safeguarding their future reproductive health and overall well-being.

## Appendices

Questionnaire

Demographic Information Gender • Male • Female Age (years) • 18-25 years • 26-35 years • 36-45 years • 46-55 years • 56 or more years Marital status • Single • Married • Divorced • Widow Educational level • Less than high school • High school • Diploma • Bachelor's • Postgraduate degree (Master's, PhD) Occupation • Employee • Unemployed • Student • Housewife Questionnaire Do you have children? • Yes • No Awareness and Knowledge of Undescended Testis At which age does undescended testis arise? • Since birth • Adolescent age • I don't know Does undescended testis affect the testicular function? • Yes • No • I don't know Does undescended testis have any complications? • Yes • No • I don't know What is the treatment of undescended testis? • Oral medication • Hormone therapy • Surgical intervention • Does not need treatment • I don't know What is the optimum time for surgery? • After birth • First five months • Six months to one year • One year and more • I don't know Is there any benefit from early treatment? • Yes • No • I don't know Do you think testicular atrophy is a potential harm of delaying the intervention? • Yes • No Do you think infertility is a potential harm of delaying the intervention? • Yes • No Do you think testicular torsion is a potential harm of delaying the intervention? • Yes • No Do you think malignancy risk is a potential harm of delaying the intervention? • Yes • No Recommendations Regarding Undescended Testis Would you recommend other parents to have their child screened for undescended testis? • Yes, definitely • Probably yes • Not sure • Probably not • No, definitely not What factors would influence your recommendation to other parents for screening their child for undescended testis? (Select all that apply) • Potential long-term consequences of untreated undescended testis • Availability of effective treatment options • Personal experience or knowledge of someone with undescended testis • Advice from healthcare professionals • Other (please specify) How important do you think early detection and treatment of undescended testis is for a child's overall health and well-being? • Extremely important • Very important • Moderately important • Slightly important • Not important at all If you had a friend or family member whose child had undescended testis, would you encourage them to seek medical evaluation and treatment? • Yes, without hesitation • Yes, but with some reservations • Not sure • No, probably not • No, definitely not How confident are you in your knowledge about undescended testis and its potential consequences? • Very confident • Somewhat confident • Neutral • Somewhat unsure • Very unsure
 
